# Thermodynamic limits for simultaneous energy harvesting from the hot sun and cold outer space

**DOI:** 10.1038/s41377-020-0296-x

**Published:** 2020-04-24

**Authors:** Wei Li, Siddharth Buddhiraju, Shanhui Fan

**Affiliations:** Department of Electrical Engineering, Ginzton Laboratory, Stanford University, Stanford, CA 94305 USA

**Keywords:** Solar energy and photovoltaic technology, Green photonics

## Abstract

The sun and outer space are two of the most important fundamental thermodynamic resources for renewable energy harvesting. A significant amount of work has focused on understanding the fundamental limit of energy harvesting from the sun. More recently, there have been several theoretical analyses of the fundamental limit of energy harvesting from outer space. However, far less is understood about the fundamental limits of simultaneous energy harvesting from both the sun and outer space. Here, we consider and introduce various schemes that are capable of simultaneous energy harvesting and elucidate the fundamental thermodynamic limits of these schemes. We show that the theoretical limits can far exceed the previously established limit associated with utilizing only one thermodynamic resource. Our results highlight the significant potential of simultaneous energy harvesting and indicate new fundamental opportunities for improving the efficiency of energy harvesting systems.

## Energy harvesting: trapping the two-way flow

Energy harvesting systems that combine capturing incoming solar energy with exploiting the emission of thermal energy outwards into space offer possibilities for improving our ability to generate power from the natural and freely available energy flows on Earth. Shanhui Fan and colleagues at Stanford University, USA, explore the theoretical limits of such simultaneous energy harvesting processes. While the theoretical efficiency of trapping incoming solar energy has been extensively studied, much less attention has been given to ‘radiative cooling’ systems, such as devices that extract energy from the dissipation of heat outwards towards the night sky. Interest in harnessing that previously neglected energy flow, however, is steadily increasing. The research team’s theoretical calculations suggest that schemes exploiting energy flow in both directions should be explored to enhance our use of the energy gradients available to us.

## Introduction

The sun at 6000 K is the most important thermodynamic resource for human beings on earth. A significant amount of current renewable energy research is focused on harvesting energy from the sun. Examples include photovoltaic systems that convert solar energy to electricity and solar thermal panels that convert solar energy to thermal energy. The fundamental limits of the efficiency of solar energy harvesting have been extensively studied^1–12^.

In addition to the sun, in recent years, there has been increasing interest in utilizing outer space at 3 K as a thermodynamic resource. In particular, substantial progress has been made in radiative cooling^13–32^, where heat from the earth at 300 K is dissipated in the form of thermal radiation to outer space. With such a temperature gradient, it is possible to set up a heat engine to extract work from the temperature gradient between the ambient environment and outer space^33–37^. Consequently, there have been several theoretical analyses on the amount of work that in principle can be extracted from such outgoing radiative heat flux^33–35,38–41^.

Thus far, most of the existing literature has focused on harvesting only one of the two thermodynamic resources. To maximize the capability of renewable energy harvesting technologies, it is important to be able to simultaneously harvest energy from these two thermodynamic resources. Recently, a tandem system that spectrally separates solar absorption and outgoing thermal radiation into a top infrared transparent solar absorber and a bottom radiative cooler was demonstrated for simultaneous solar heating and radiative cooling^42^. There have also been efforts aiming to improve solar cell performance through radiative cooling^27,43–48^. However, for energy harvesting purposes, the full potential and the fundamental limits of systems that can simultaneously utilize the sun and outer space have not been elucidated.

In this paper, we consider and introduce various schemes that are capable of simultaneous energy harvesting using both the sun and outer space and determine the fundamental thermodynamic limits of these schemes. We show that many of these schemes allow one to generate more power from solar energy harvesting or harvesting of the outgoing thermal radiation alone, with theoretical limits far beyond the well-established solar energy harvesting limits. Our results highlight the significant potential of simultaneous energy harvesting, indicate new fundamental opportunities and provide guidance for improving the efficiency of energy harvesting systems.

## Results

For all the calculations in the manuscript, we make the assumptions that the temperatures of the sun, outer space, and the ambient environment are 6000 K, 3 K, and 300 K, respectively. The atmosphere is not considered in the calculation. These assumptions are consistent with all of the previously established theoretical limits for solar energy harvesting^1,2,9,49^ and harvesting of outgoing thermal radiation^33–35^.

**Fig. 1:**
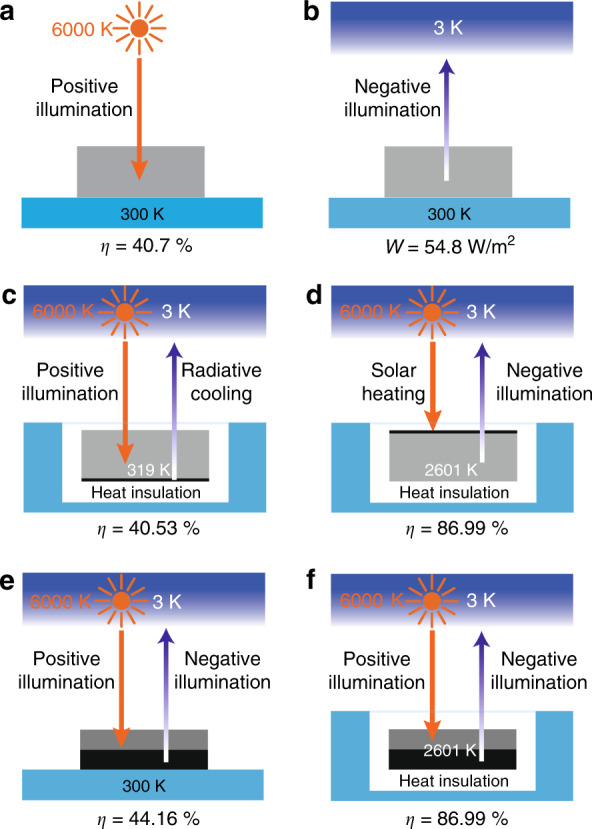
Shockley–Queisser limit for simultaneous energy harvesting. **a** Schematic of a solar cell working under positive illumination conditions. The cell is maintained at a temperature of 300 K. **b** Schematic of a solar cell working under negative illumination conditions. The cell is maintained at a temperature of 300 K. **c** Schematic of a solar cell working under positive illumination conditions combined with radiative cooling. The cell is thermally insulated from the ambient environment, and the temperature is purely determined by the radiative heat exchange, with an equilibrium temperature of 319 K. **d** Schematic of a solar cell working under negative illumination conditions combined with solar heating. The cell is thermally insulated from the ambient environment, and the temperature is purely determined by the radiative heat exchange, with an equilibrium temperature of 2601 K. **e** Schematic of a combined system that utilizes both positive illumination and negative illumination. The system is maintained at a temperature of 300 K. **f** Schematic of a combined system that utilizes both positive illumination and negative illumination. The system is thermally insulated from the ambient environment, and the temperature is purely determined by the radiative heat exchange, with an equilibrium temperature of 2601 K

We assume direct, unconcentrated sunlight. Most of the setups considered in this paper consist of a solar absorber and a thermal emitter placed on top of each other. (e.g., see Fig. 1e). We assume that the absorber and the emitter have the following *angle-selective characteristics*: the solar absorber exhibits absorption only for incident sunlight within the solid angle of the sun and exhibits no absorption outside the solid angle of the sun. Hence, the view factor of the solar absorber towards the sun is *f* = 2.18 × 10^−5^. Such an angle restriction ensures that the solar absorber performs at the same theoretical limit as the limit for full concentration^1,7,50,51^. The thermal emitter is assumed to have zero emissivity/absorptivity within, and unity emissivity outside, the solid angle of the sun. Hence, the view factor of the thermal emitter with respect to outer space is 1 − *f*. Consequently, there is no radiative exchange between the solar absorber and the thermal emitter. Moreover, both the solar absorber and thermal emitter can utilize the entire solar and thermal radiation spectrum for energy harvesting purposes.

In this paper, we examine a number of energy harvesting systems. These systems incorporate devices that extract power from either incoming or outgoing thermal radiation. We define a device that extracts power from incoming photon flux as a device that operates under *positive illumination*. An example of such a device is a typical solar cell facing the sun. Similarly, we define a device that extracts power from outgoing photon flux as a device that operates under *negative illumination*. An example of such a device that was recently demonstrated^34,37^ is a narrow-bandgap photodiode facing the night sky.

Conventionally, when solar energy harvesting is discussed, the performance is typically measured in terms of an efficiency^1,2,9,49^ defined as the ratio of the extracted work to the incident power from sunlight. On the other hand, in the case of energy harvesting from outgoing thermal radiation, the performance is usually measured in terms of power density^33–35^ rather than efficiency. The reason for this difference is as follows: in the solar energy harvesting case, the incident sunlight power, which is the heat input, is kept fixed. In contrast, in the case of harvesting outgoing thermal radiation, the heat input is through conduction from the ambient environment and varies according to the operating condition of the device. While one can define an efficiency for each device, it becomes more difficult to compare the efficiencies of different devices. In our case of simultaneous energy harvesting, we follow the convention for solar energy harvesting and define the efficiency as the ratio of the extracted work from the system to the incident sunlight power. Since some of our setups involve multiple work extraction processes from three temperatures, i.e., the hot sun, cold outer space and the earth, the efficiency is not constrained by the two-temperature Carnot limit between the sun and the earth, since in these schemes, there is an extra heat input from the earth.

### Shockley–Queisser limit

We start with a discussion of energy harvesting from a single-junction photovoltaic cell consisting of a semiconductor photodiode. When facing a hot object such as the sun, i.e., under positive illumination, such a photodiode can absorb a net in-flux of photons with an energy above its bandgap to produce electrical power. The theoretical efficiency limit of a single-junction cell for harvesting incoming radiation from the sun (Fig. 1a) is well established as the Shockley–Queisser limit^1^. Under full concentration or angle restricted absorption and emission, a maximum efficiency of 40.7% can be obtained at an optimal bandgap energy of 1.08 eV when the cell is maintained at a temperature of 300 K. On the other hand, it was recently shown that when the diode faces a cold object, i.e., under negative illumination^34^, electrical power can also be extracted from the diode. In ref. ^34^, the corresponding Shockley–Queisser limit of a single-junction cell for harvesting energy using outgoing radiation to outer space (Fig. 1b) was derived^34^, resulting in a maximum power density of 54.8 Wm^−2^. Building upon these previous works on energy harvesting from either positive or negative illumination, in this section, we consider various approaches of energy harvesting from both positive and negative illumination simultaneously involving the use of a photodiode.

In the first approach, we consider the configuration shown in Fig. 1c, where we combine a single-junction cell working under positive illumination conditions with radiative cooling. In this setup, the cell is assumed to have unity absorption above the bandgap to produce a photocurrent and zero absorption below the bandgap. A blackbody radiative cooling layer is placed on the bottom side of the cell. Both the cell and the radiative cooling layer exhibit the angle-selective characteristics discussed earlier. Here, we assume ideal thermal contact between the radiative cooler and the cell so that they have the same temperature *T*_c_. In contrast to the typical Shockley-Queisser limit, where the cell is assumed to be in good thermal contact with the ambient environment at 300 K, here, we assume that the cell is thermally insulated from the ambient environment. The temperature of the cell *T*_c_ is instead determined only by its radiative heat exchange with the sun (*T*_s_ = 6000 K) and the radiative heat exchange of the radiative cooler with outer space (*T*_o_ = 3 K). To compute the efficiency of the system, we perform a detailed balance analysis to determine the electrical power density $\dot W$ produced by the cell:

1 $$\dot W = qV{\int}_{E_{\mathrm{G}}}^\infty {dE\,\rho \left( E \right)\left( {f \cdot \theta \left( {E,T_{\mathrm{S}},0} \right) - f\cdot\theta \left( {E,T_{\mathrm{c}},V} \right)} \right)}$$

The electrical power density $\dot W$ is maximized with respect to the band gap energy of the semiconductor *E*_G_ and the voltage on the cell *V*. In Eq. (1), *q* is the unit electric charge, $\rho \left( E \right) = \frac{{E^2}}{{4\pi ^2\hbar ^3c^2}}$ is the photon density of states at photon energy E, $\hbar$ is the reduced Planck’s constant, *c* is the speed of light in vacuum. $\theta \left( {E,T,V} \right) = 1/\left( {\frac{{\exp \left( {E\;-\;qV} \right)}}{{kT}} - 1} \right)$ is the number of photons in a mode at energy *E*, and *k* is the Boltzmann constant. We solve for the temperature of the cell, *T*_c_, using the thermal balance equation:

2 $$P_{{\mathrm{in}}}^{{\mathrm{cell}}} + P_{{\mathrm{in}}}^{{\mathrm{cooling}}} = P_{{\mathrm{out}}}^{{\mathrm{cell}}} + P_{{\mathrm{out}}}^{{\mathrm{cooling}}}$$

Here, $P_{{\mathrm{in}}}^{{\mathrm{cell}}} = {\int}_{E_{\mathrm{G}}}^\infty {dE\left( {E - qV} \right)\rho \left( E \right)\left( {f \cdot \theta \left( {E,T_{\mathrm{S}},0} \right)} \right)}$ is the heat generated in the cell by absorbing photons from the sun with an energy larger than the cell bandgap *E*_G_. $P_{{\mathrm{in}}}^{{\mathrm{cooling}}} = {\int}_0^\infty {dE\,E\,\rho \left( E \right)\left( {\left( {1 - f} \right) \cdot \theta \left( {E,T_{\mathrm{o}},0} \right)} \right)}$ is the heat received by the bottom radiative cooling layer from outer space at *T*_o_. $P_{{\mathrm{out}}}^{{\mathrm{cell}}} = {\int}_{E_{\mathrm{G}}}^\infty {dE\left( {E - qV} \right)\rho \left( E \right)\left( {f \cdot \theta \left( {E,T_{\mathrm{c}},V} \right)} \right)}$ is the heat dissipation of the cell through re-emission at temperature *T*_c_ and voltage *V*. Lastly, $P_{{\mathrm{out}}}^{{\mathrm{cooling}}} = {\int}_0^\infty {dE\,E\,\rho \left( E \right)\left( {\left( {1 - f} \right) \cdot \theta \left( {E,T_{\mathrm{c}},0} \right)} \right)}$ is the heat dissipation of the bottom radiative cooling layer through thermal radiation.

The maximum efficiency of this system is 40.53% when the cell bandgap energy *E*_G_ is 1.13 eV. This efficiency is slightly lower than the Shockley-Queisser limit, as the cell temperature *T*_c_ in the presence of radiative cooling is 319 K, which is larger than the ambient temperature of 300 K. Such a reduction in efficiency due to an increased cell temperature is well known^52^. However, this efficiency can be further improved if one allows the radiative cooling layer to have a larger area than the cell. In addition, for cells with a bandgap energy larger than 1.4 eV, such a setup can achieve an efficiency higher than the efficiency of the same single-junction cell operating by itself, as shown in Fig. 1a.

As a second approach, we consider a single-junction cell working under negative illumination conditions^34,37^ and combine the cell with solar heating (Fig. 1d). We add a solar absorbing layer on the top side of the cell (Fig. 1d). The solar absorbing layer and the cell (now serving as the thermal emitter) exhibit the angle-selective characteristics discussed above. Again, we assume the cell to be thermally insulated from the ambient environment and that the temperature of the cell *T*_c_ is determined only by its radiative heat exchanges with the sun and outer space. We perform a detailed balance and thermal balance analysis to compute the power output from the cell:

3 $$\dot W = qV{\int}_{E_{\mathrm{G}}}^\infty {dE\,\rho \left( E \right)\left( {(1 - f) \cdot \theta \left( {E,T_{\mathrm{o}},0} \right) - \left( {1 - f} \right) \cdot \theta \left( {E,T_{\mathrm{c}},V} \right)} \right)}$$

where the temperature of the cell, *T*_c_, is determined by the heat balance equation

4 $$P_{{\mathrm{in}}}^{{\mathrm{cell}}} + P_{{\mathrm{in}}}^{{\mathrm{heating}}} = P_{{\mathrm{out}}}^{{\mathrm{cell}}} + P_{{\mathrm{out}}}^{{\mathrm{heating}}}$$

In this case, $P_{{\mathrm{in}}}^{{\mathrm{cell}}} = {\int}_{E_{\mathrm{G}}}^\infty {dE\left( {E - qV} \right)\rho \left( E \right)\left( {\left( {1 - f} \right) \cdot \theta \left( {E,T_{\mathrm{o}},0} \right)} \right)}$ is the heat generation in the cell by absorbing photons from outer space with an energy larger than the cell bandgap *E*_G_. $P_{{\mathrm{in}}}^{{\mathrm{heating}}} = {\int}_0^\infty {dE\,qE\,\rho \left( E \right)\left( {f \cdot \theta \left( {E,T_{\mathrm{S}},0} \right)} \right)}$ is the heat received by the top solar absorbing layer from the sun. $P_{{\mathrm{out}}}^{{\mathrm{cell}}} = \mathop {\int }\nolimits_{E_{\mathrm{G}}}^\infty dE\left( {E - qV} \right)\rho \left( E \right)\left( {\left( {1 - f} \right) \cdot \theta \left( {E,T_{\mathrm{c}},V} \right)} \right)$ is the heat dissipation of the cell through re-emission at temperature *T*_c_ and voltage *V*. $P_{{\mathrm{out}}}^{{\mathrm{heating}}} = {\int}_0^\infty {dE\,qE\,\rho \left( E \right)\left( {f \cdot \theta \left( {E,T_{\mathrm{c}},0} \right)} \right)}$ is the heat dissipation of the top solar absorbing layer through thermal radiation.

From Eqs. (3) and (4), the cell temperature is determined to be *T*_c _= 2601 K. The maximum efficiency of this system reaches 86.99%. This value is much higher than the value in the positive illumination case of Fig. 1c because in this system, the incoming thermal radiation from the sun is used to elevate the cell temperature, which in turn significantly boosts the outgoing thermal radiation and the power generation. This result suggests a potential alternative approach to realize high-efficiency solar energy conversion.

In the third approach, we combine a top cell working under positive illumination conditions with a bottom cell working under negative illumination conditions (Fig. 1e). The top and bottom cells now operate as the solar absorber and thermal emitter with the angle-selective characteristics discussed earlier. The system is kept in good thermal contact with the 300 K ambient environment. The total efficiency, in this case, reaches 44.16%, which is higher than the typical Shockley–Queisser limit of 40.7% due to the additional contribution from the cell working under negative illumination conditions.

As the fourth approach, shown in Fig. 1f, we consider a system similar to the system in the third approach, with the difference that the cell is thermally insulated from the ambient environment and its temperature is determined only by its radiative heat exchanges. In this case, the maximum efficiency of the combined cell reaches 86.99%, similar to the case shown in Fig. 1d.

### Multicolor limit

**Fig. 2:**
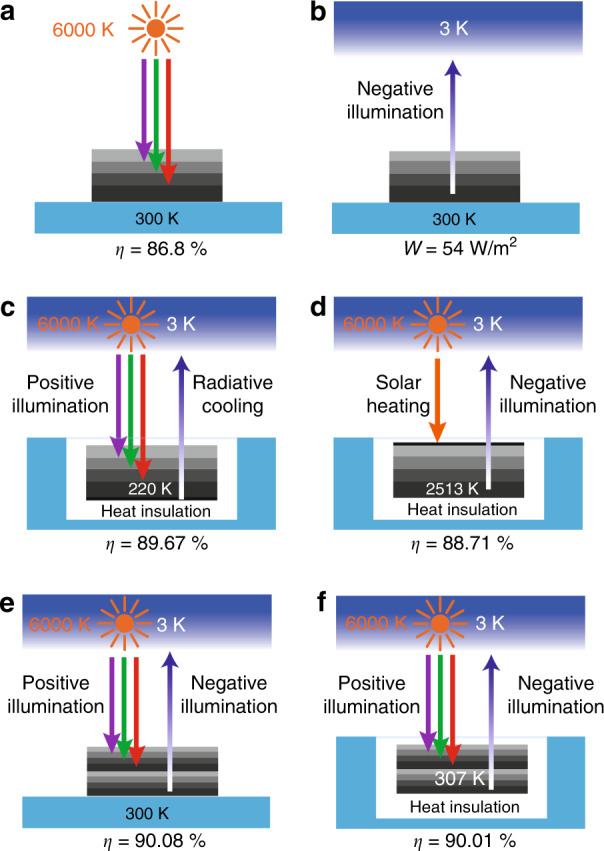
Multicolor limit for simultaneous energy harvesting. **a** Schematic of a multijunction cell working under positive illumination conditions. The multicolor limit of energy harvesting from the sun is reached with an infinite number of cells. **b** Schematic of a multijunction cell working under negative illumination conditions. The multicolor limit of energy harvesting from outer space is reached with an infinite number of cells. **c** Schematic of a multijunction cell working under positive illumination conditions combined with radiative cooling. The cell is thermally insulated from the ambient environment, and the temperature is purely determined by the radiative heat exchange, with an equilibrium temperature of 220 K. **d** Schematic of a multijunction cell working under negative illumination conditions combined with solar heating. The cell is thermally insulated from the ambient environment, and the temperature is purely determined by the radiative heat exchange, with an equilibrium temperature of 2513 K. **e** Schematic of a combined multijunction system that utilizes both positive illumination and negative illumination. The system is maintained at 300 K. **f** Schematic of a combined multijunction system that utilizes both positive illumination and negative illumination. The system is thermally insulated from the ambient environment, and the temperature is purely determined by the radiative heat exchange, with an equilibrium temperature of 307 K

For solar energy conversion, it is known that efficiencies higher than the efficiency of the single-junction cell setup of Fig. 1a are possible through the idealized multijunction cell system shown in Fig. 2a. This system consists of a stack of semiconductor photodiodes, with the cells nearer to the top of the stack having an increasingly larger bandgap (Fig. 2a). In the limit of an infinite number of cells, an efficiency of 86.8%, typically referred to as the multicolor limit, can be achieved^8,53^ for such a cell facing the sun and maintained at the ambient temperature of 300 K. Similarly, one can use the same multijunction cell configuration for energy harvesting from outer space (Fig. 2b), where the multicolor limit of 55 Wm^−2^ is reached with the use of an infinite number of bandgaps^34,35^ when the cell is at the ambient temperature of 300 K. Here, we follow the previous section and apply the same four approaches to simultaneously utilize the sun and outer space in a multijunction cell system.

In the first approach, we consider a multijunction cell working under positive illumination conditions and combine the cell with radiative cooling by adding a bottom radiative cooling layer (Fig. 2c). The cell and the bottom radiative cooling layer exhibit the same angle-selective characteristics as in Fig. 1c. We perform a detailed balance and thermal balance analysis to compute the maximum efficiency by following a procedure similar to Eqs. (1) and (2). The only difference is that here the operating voltage *V*(*E*) of each photodiode is optimized as a function of the photon energy *E* (see Methods). The maximum efficiency of this system is 89.67%, with an optimal cell temperature *T*_c_ of 220 K. In contrast to the case of a single-junction cell with radiative cooling (Fig. 1c), where the cell temperature does not decrease below the ambient temperature, here, the cell temperature can be significantly below the ambient temperature, demonstrating the efficacy of radiative cooling. This efficiency significantly exceeds the previously established multicolor limit of 86.8% for solar energy harvesting.

We then consider a multijunction cell working under negative illumination conditions and combine the cell with solar heating by adding a top solar absorber (Fig. 2d). The solar absorber and the cell exhibit the angle-selective characteristics discussed earlier. Similar to the setup in Fig. 1d, the cell is heated by the top heating layer when the cell is performing negative illumination. The maximum efficiency of this system reaches 88.71%. This efficiency also significantly exceeds the previously established 86.8% multicolor limit for solar energy harvesting. Compared to the previously discussed single-junction cell working under negative illumination conditions and solar heating with an efficiency of 86.99%, the multijunction cell configuration further improves the efficiency.

In the third case, we combine a multijunction cell working under positive illumination conditions and a multijunction cell working under negative illumination conditions (Fig. 2e). The cell is maintained at the ambient temperature of 300 K. The top positive illumination cell and bottom negative illumination cell exhibit the angle-selective characteristics discussed earlier. The total efficiency, in this case, reaches 90.08%, which is significantly higher than the typical multicolor limit of 86.8%.

Finally, we consider a system that is similar to the system in Fig. 2e but thermally insulated from the ambient environment, and its temperature is determined only by its heat exchanges with the sun and outer space (Fig. 2f). In this case, the maximum efficiency of the combined cell reaches 90.01%, similar to the case of Fig. 2e. These results indicate that the multijunction cell system has significant potential for simultaneously utilizing both the sun and outer space to improve the energy conversion efficiency.

### Blackbody limit

**Fig. 3:**
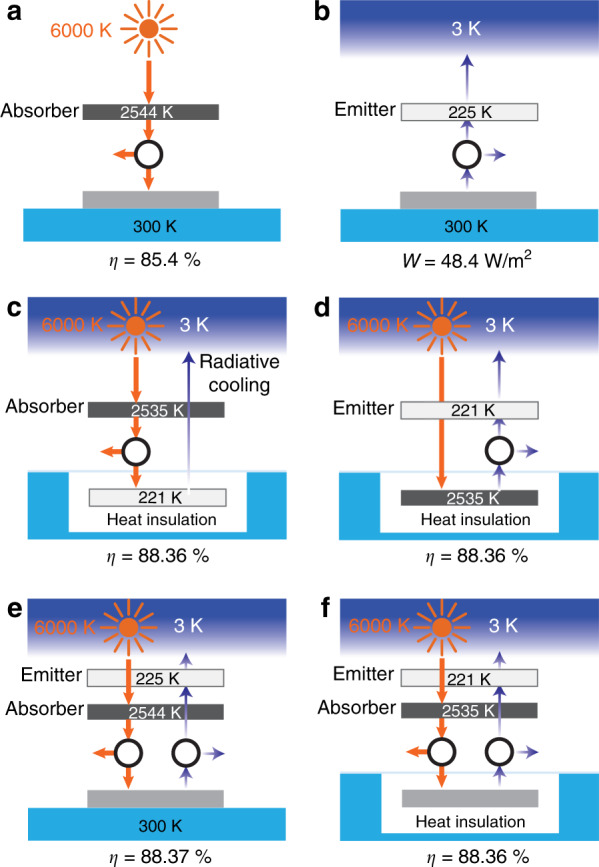
Blackbody limit for simultaneous energy harvesting. **a** Schematic of a standard setup implemented to reach the blackbody limit of energy harvesting from the sun. A Carnot engine is placed between the intermediate absorber and the cell. The cell is maintained at 300 K. **b** Schematic of a standard setup implemented to reach the blackbody limit of energy harvesting from outer space. A Carnot engine is placed between the intermediate emitter and the cell. The cell is maintained at 300 K. **c** Schematic of a thermophotovoltaic system working under positive illumination conditions combined with radiative cooling. The cell is thermally insulated from the ambient environment, and the temperature is purely determined by the radiative heat exchange. **d** Schematic of a thermophotovoltaic system working under negative illumination conditions combined with solar heating. The cell is thermally insulated from the ambient environment, and the temperature is purely determined by the radiative heat exchange. **e** Schematic of a thermophotovoltaic system combining both positive illumination and negative illumination. The cell is maintained at 300 K. **f** Schematic of a thermophotovoltaic system combining both positive illumination and negative illumination. The cell is thermally insulated from the ambient environment, and the temperature is purely determined by the radiative heat exchange

It is well known that in solar energy harvesting, a solar thermophotovoltaic system^10,54^ can have a theoretical efficiency significantly beyond the single-junction Shockley–Queisser limit. The limiting efficiency of a solar thermophotovoltaic system, typically referred to as the blackbody limit^10^, is derived using the setup shown in Fig. 3a. This setup consists of an intermediate solar absorber at a steady-state temperature of *T*_a_ with a net radiative heat exchange from the sun. A Carnot engine is placed between the solar absorber and a heat sink at 300 K to extract work. The maximum efficiency of such a system reaches 85.4% when the intermediate absorber temperature is 2544 K. Recently, a similar thermophotovoltaic system working under negative illumination conditions was proposed to harvest energy from outer space^33^ (Fig. 3b). In this system, an intermediate blackbody emitter facing outer space lowers its temperature through radiative cooling. A Carnot engine is placed between the heat sink and the intermediate emitter to extract work. The maximum power density extracted by such a system is 48.4 Wm^−2^ (ref. ^33^). Building upon these previous works in thermophotovoltaic systems, here, we discuss various approaches in which one can use the thermophotovoltaic system to simultaneously harvest energy from the sun and outer space.

In the first approach, we consider a system combining a solar thermophotovoltaic system working under positive illumination conditions with radiative cooling (Fig. 3c). The system consists of an intermediate solar absorber at a steady-state temperature of *T*_a_, with a net radiative heat flux exchange from the sun. A Carnot engine is placed between the solar absorber and a heat sink to extract work. The work extracted by this system is:

5 $$\dot W = f\sigma \left( {T_{\mathrm{s}}^4 - T_{\mathrm{a}}^4} \right)\left( {1 - \frac{{T_{\mathrm{c}}}}{{T_{\mathrm{a}}}}} \right)$$

In contrast to the typical solar thermophotovoltaic system (Fig. 3a), where the heat sink is maintained at the ambient temperature of 300 K, in this system, we assume that the heat sink is a thermal emitter performing radiative cooling. Both the solar absorber and thermal emitter exhibit angle-selective characteristics, as discussed earlier. The temperature of the thermal emitter *T*_c_ is determined by a thermal balance between the heat received by the thermal emitter from the Carnot engine and the heat dissipation of the thermal emitter to outer space through radiative cooling:

6 $$f\sigma \left( {T_{\mathrm{s}}^4 - T_{\mathrm{a}}^4} \right)\left( {\frac{{T_{\mathrm{c}}}}{{T_{\mathrm{a}}}}} \right) = \left( {1 - f} \right)\sigma \left( {T_{\mathrm{c}}^4 - T_{\mathrm{o}}^4} \right)$$

The system reaches a maximum efficiency of $\eta = \frac{{\dot W}}{{f\sigma T_{\mathrm{s}}^4}} = 88.36\%$ when the solar absorber is at the optimal temperature of *T*_a_ = 2535 K and the thermal emitter has a temperature of *T*_c_ = 221 K. The efficiency is significantly improved compared to the typical 85.4% efficiency of the solar thermophotovoltaic system, since the temperature of the thermal emitter can drop below the ambient temperature due to radiative cooling.

In the second approach, we consider a thermophotovoltaic system operating under negative illumination conditions and combine the system with solar heating (Fig. 3d). The system consists of an intermediate emitter with a temperature of *T*_e_ and a solar-heated heat sink with a temperature of *T*_c_. A Carnot engine is placed between the emitter and a heat sink to extract work. Similar to the first approach, this system reaches a maximum efficiency of $\eta = \frac{{\dot W}}{{f\sigma T_{\mathrm{s}}^4}} = 88.36\%$ when the sink temperature *T*_c_ is 2535 K and the emitter temperature *T*_e_ is 221 K.

We then consider a system that combines both positive illumination and negative illumination, as shown in Fig. 3e. The system consists of an intermediate blackbody absorber at a steady-state temperature of *T*_a_ with a net radiative heat flux exchange from the sun and an intermediate blackbody emitter at a steady-state temperature of *T*_e_ with a net radiative heat flux exchange from outer space. Two Carnot engines are placed between the blackbody emitter and the heat sink and between the blackbody emitter and the heat sink to extract work. The heat sink is maintained at the ambient temperature of 300 K. In this case, the system reaches a maximum efficiency of $\eta = \frac{{\dot W}}{{f\sigma T_{\mathrm{s}}^4}} = 88.37\%$ when the intermediate absorber temperature *T*_a_ is 2544 K and the emitter temperature *T*_e_ is 225 K. The efficiency is significantly improved compared to the typical 85.4% efficiency of the solar thermophotovoltaic system due to the extra power extracted from negative illumination.

Finally, we consider a system similar to the system in Fig. 3e, but the heat sink is thermally insulated from the ambient environment and its temperature *T*_c_ is determined only by its heat exchanges with the two Carnot engines (Fig. 3f). In this case, the system reaches a maximum efficiency of $\eta = \frac{{\dot W}}{{f\sigma T_{\mathrm{s}}^4}} = 88.36\%$ when the intermediate absorber temperature *T*_a_ is 2535 K and the emitter temperature *T*_e_ is 221 K. In this case, the maximum efficiency is independent of the cell temperature *T*_c_. The efficiency is again significantly improved compared to the typical 85.4% efficiency of the solar thermophotovoltaic system. These results indicate that thermophotovoltaic systems have significant potential for simultaneously utilizing both the sun and outer space for energy harvesting.

### Landsberg limit

**Fig. 4:**
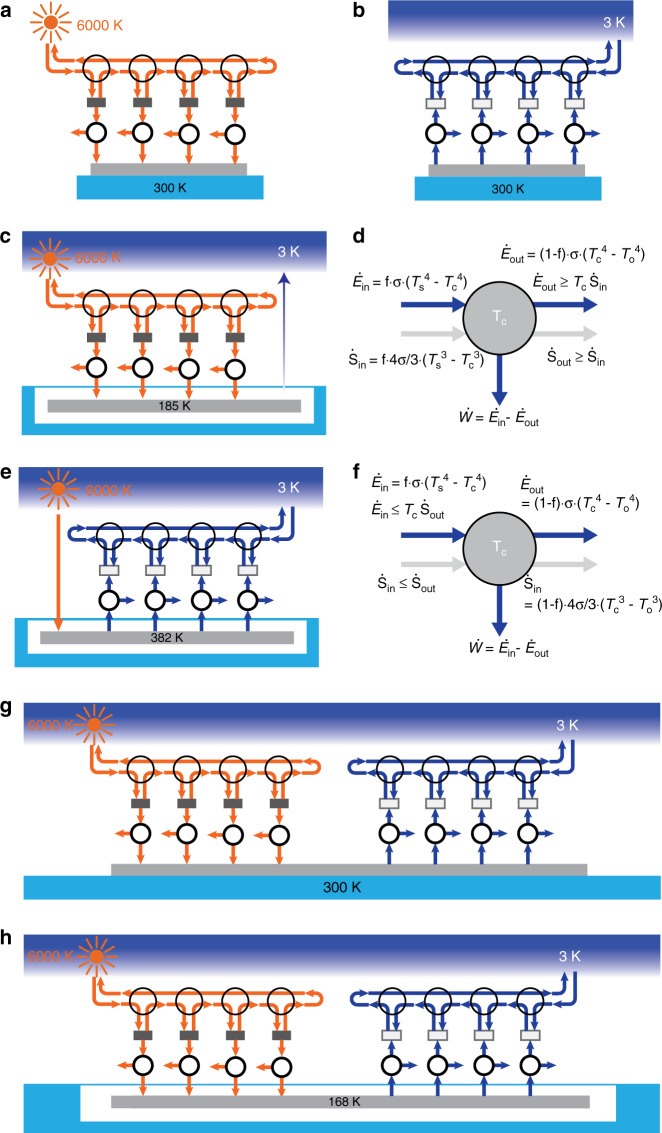
Landsberg limit for simultaneous energy harvesting. **a** Schematic of a standard setup implemented to reach the Landsberg limit under positive illumination conditions. The larger open circles represent circulators that allow reciprocity breaking to enable unidirectionality of the radiative heat flow. In the limit of an infinite number of circulators, these engines reach the Landsberg limits. **b** Schematic of a setup implemented to reach the Landsberg limit under negative illumination conditions. **c** Schematic of a Landsberg limit system combined with radiative cooling. The cell is thermally insulated from the ambient environment, and the temperature is determined by the radiative heat exchange, with an equilibrium temperature of 185 K. **d** Schematic used to compute the maximum power of the system in **c**. **e** Schematic of a Landsberg limit system working under negative illumination conditions combined with solar heating. The cell is thermally insulated from the ambient environment, and the temperature is determined by the radiative heat exchange, with an equilibrium temperature of 382 K. **f** Schematic used to compute the maximum power of the system in **e**. **g** Schematic of a Landsberg limit system combining both positive and negative illumination. The cell is in good thermal contact with the ambient environment at 300 K. **h** Schematic of a Landsberg limit system combining both positive and negative illumination. The cell is thermally insulated from the ambient environment, and the temperature is determined by the radiative heat exchange, with an equilibrium temperature of 168 K

In the previous sections, we focused on thermal radiation energy harvesting systems that obey Lorentz reciprocity^55,56^. Lorentz reciprocity applies to any material that is described by symmetric permittivity and permeability tensors and in practice applies to most materials typically considered in thermal radiation. One important consequence of Lorentz reciprocity is Kirchhoff’s law, which states that the angular spectral absorptivity and emissivity must be equal to each other^57^. Therefore, in a reciprocal solar energy harvesting system, a good solar absorber is also a good thermal emitter in the same spectral and angular range, thus inevitably re-emitting a part of the absorbed energy to the sun and reducing its efficiency. It is known that breaking Lorentz reciprocity can lead to theoretical efficiencies exceeding the efficiency of reciprocal systems^49^. For positive illumination, the ultimate limit for solar energy harvesting, known as the Landsberg limit^49^, corresponds to a maximum efficiency of 93.3%. The proposed physical realization designed to reach this Landsberg limit^58,59^ (Fig. 4a) consists of a sequence of intermediate absorbers whose temperatures gradually change from the sun temperature *T*_s_ = 6000 K to the heat sink temperature *T*_c_ = 300 K. Carnot engines operate between each absorber and the heat sink to extract work. Unlike the reciprocal blackbody system where the emission from the absorber is directly sent back to the sun, here, the emission from each absorber is rerouted to the next adjacent absorber at a lower temperature with the use of a circulator^58,59^. The last absorber at the lowest temperature emits back to the sun. Recently, a Landsberg setup working under negative illumination conditions was also proposed to perform energy harvesting from outer space^35^ (Fig. 4b). The maximum power extracted in such a system is 153.1 Wm^-2^. Similar to the previous sections, here, we present four Landsberg systems to simultaneously utilize the sun and outer space and compare the performance with the existing limits.

In the first approach, we consider a system combining the Landsberg system working under positive illumination conditions with radiative cooling (Fig. 4c). In this system, the heat sink is thermally insulated from the ambient environment, and its temperature *T*_c_ is determined only by its heat exchanges. We perform an exergy analysis to compute the maximum work that can be extracted from this system. Exergy is the maximum fraction of the input energy flux $\dot E_{in}$ that can be extracted into work by a heat engine operating at a temperature T^60^ and can be computed as $\dot E_{{\mathrm{in}}} - T\dot S_{{\mathrm{in}}}$, where $\dot S_{{\mathrm{in}}}$ is the entropy flux. We consider a hypothetical engine as shown in Fig. 4d. The heat sink at the temperature of *T*_c_ receives heat input from the sun with a net radiative heat flux of $\dot E_{{\mathrm{in}}} = f \cdot \sigma \left( {T_{\mathrm{s}}^4 - T_{\mathrm{c}}^4} \right)$, accompanied by the corresponding net entropy flux $\dot S_{{\mathrm{in}}} = f \cdot \left( {4/3} \right)\sigma \left( {T_{\mathrm{s}}^3 - T_{\mathrm{c}}^3} \right)$. Based on the second law, the entropy rejected by the engine, $\dot S_{{\mathrm{out}}}$, must be at least as large as $\dot S_{{\mathrm{in}}}$. The upper bound on the extraction of work is reached when $\dot S_{{\mathrm{out}}} = \dot S_{{\mathrm{in}}}$. As a result, the exergy of the incoming heat flow, or the maximum amount of work that can be extracted from this setup at the temperature of *T*_c_, is:

7 $$\dot W = \dot E_{{\mathrm{in}}} - \dot E_{{\mathrm{out}}} = \dot E_{{\mathrm{in}}} - T_{\mathrm{c}}\dot S_{{\mathrm{in}}} = f \cdot \sigma \left( {T_{\mathrm{s}}^4 - T_{\mathrm{c}}^4} \right) - T_{\mathrm{c}} \cdot f \cdot \left( {4/3} \right)\sigma \left( {T_{\mathrm{s}}^3 - T_{\mathrm{c}}^3} \right)$$

The heat sink temperature *T*_c_ can be determined by the heat balance between the heat dissipation from the engine and the radiative cooling to outer space:

8 $$\dot E_{{\mathrm{out}}} = \left( {1 - f} \right) \cdot \sigma \left( {T_{\mathrm{c}}^4 - T_{\mathrm{o}}^4} \right)$$

The system reaches a maximum efficiency of $\eta = \frac{{\dot W}}{{f\sigma T_{\mathrm{s}}^4}} = 95.9\%$ with the optimal heat sink temperature *T*_c_ of 184.5 K. The efficiency is significantly improved compared to the typical 93.3% efficiency of the Landsberg limit.

Similarly, we consider a Landsberg system working under negative illumination conditions and combine the system with solar heating (Fig. 4e). In this case, the heat sink is thermally insulated from the ambient environment, and its temperature *T*_c_ is determined only by its radiative heat exchanges. To compute the maximum work, we consider a hypothetical engine as shown in Fig. 4f. The engine at the temperature of *T*_c_ has an outgoing heat flux equal to the net radiative heat flux between the ambient and outer space $\dot E_{{\mathrm{out}}} = \left( {1 - f} \right) \cdot \sigma \left( {T_{\mathrm{c}}^4 - T_{\mathrm{o}}^4} \right)$, accompanied by the corresponding net entropy flux $\dot S_{{\mathrm{out}}} = \left( {1 - f} \right) \cdot \left( {4/3} \right)\sigma \left( {T_{\mathrm{c}}^3 - T_{\mathrm{o}}^3} \right)$. Based on the second law, the input entropy $\dot S_{{\mathrm{in}}}$ must at most be equal to $\dot S_{{\mathrm{out}}}$. The upper bound on the extraction of work is reached when $\dot S_{{\mathrm{in}}} = \dot S_{{\mathrm{out}}}$. As a result, the exergy of the incoming heat flow, or the maximum amount of work that can be extracted, is:

9 $$\dot W = \dot E_{{\mathrm{in}}} - \dot E_{{\mathrm{out}}} = T_{\mathrm{c}}\dot S_{{\mathrm{out}}} - \dot E_{{\mathrm{out}}} = T_{\mathrm{c}}\left( {1 - f} \right)\left( {\frac{4}{3}} \right)\sigma \left( {T_{\mathrm{c}}^3 - T_{\mathrm{o}}^3} \right) - \left( {1 - f} \right)\sigma \left( {T_{\mathrm{c}}^4 - T_{\mathrm{o}}^4} \right)$$

*T*_c_ can be determined by the heat balance between the solar heating from the sun and the maximum heat flux drawn from the engine:

10 $$f \cdot \sigma \left( {T_{\mathrm{s}}^4 - T_{\mathrm{c}}^4} \right) = \dot E_{{\mathrm{in}}}$$

This system achieves a maximum work of $\dot W$= 400.5 Wm^−2^ and an efficiency of $\eta = \frac{{\dot W}}{{f\sigma T_{\mathrm{s}}^4}} = 25.0\%$, with a cell temperature of 381.5 K.

We then consider a Landsberg setup that combines both positive illumination and negative illumination, as shown in Fig. 4g. The cell is in good thermal contact with the ambient environment at 300 K. In this case, the maximum work that can be extracted by this system is 1648.2 Wm^−2^, corresponding to an efficiency of $\eta = \frac{{\dot W}}{{f\sigma T_{\mathrm{s}}^4}} = 102.89\%$. It should be noted that this efficiency exceeds 100% as a portion of the power is obtained through the outgoing thermal radiation from the ambient to outer space. Nevertheless, the maximum work here significantly exceeds the established limit that is achievable by using only the sun or outer space as a thermodynamic resource, highlighting the importance of simultaneous energy harvesting from the sun and the cold universe.

Finally, we consider a system that is similar to the system in Fig. 4g, but the heat sink is thermally insulated from the ambient environment and its temperature *T*_c_ is determined only by its heat exchanges. In this case, the system achieves a maximum efficiency of $\eta = \frac{{\dot W}}{{f\sigma T_{\mathrm{s}}^4}} = 97.2\%$, with an optimal heat sink temperature *T*_c_ of 167.6 K. This efficiency is also significantly higher than the Landsberg limit of 93.3%. These results indicate the significant potential of simultaneously utilizing both the sun and outer space in Landsberg setups for energy harvesting.

## Discussion

We propose a variety of schemes that are capable of simultaneous energy harvesting from both the sun and outer space and elucidate the fundamental thermodynamic limits of these schemes. The results of these limits are summarized in Table 1, which show that these schemes allow one to generate more power from solar energy harvesting or harvesting of outgoing thermal radiation alone, with theoretical limits far beyond the well-established solar energy harvesting limits.

**Table 1:** Summary of simultaneous energy harvesting systems and a comparison with systems that only utilize one resource

	Shockley–Queisser	Multicolor	Blackbody	Landsberg
Positive illumination @ 300 K	40.74%	86.8%	85.4%	93.3%
Negative illumination @ 300 K	54.8 Wm^−2^	55.0 Wm^−2^	48.4 Wm^−2^	153.1 Wm^−2^
Positive illumination + radiative cooling	40.53%	89.67%	88.36%	95.9%
Negative illumination + solar heating	86.99%	88.71%	88.36%	24.99%
Positive illumination + negative illumination	86.99%	90.01%	88.36%	97.21%
Positive illumination + negative illumination @ 300 K	44.16%	90.08%	88.37%	102.89%

Here, we present a brief discussion of the experimental progress in solar and outgoing thermal radiation energy harvesting and promising pathways to improve the efficiency of existing energy harvesting systems by combining these harvesting approaches. There is a large body of literature for solar energy harvesting. The highest efficiencies reported so far are 29.3 ± 0.7% for a single-junction cell^61^, 47.1 ± 2.6% for a multijunction cell^62^, and 6.8% for solar thermophotovoltaics^63^. There has not been an experimental demonstration of a setup similar to Fig. 4 (referred to as the Landsberg setup) that uses non-reciprocal elements. On the other hand, there have only been a few recent initial experimental demonstrations of outgoing thermal radiation energy harvesting. The reported power densities so far are 6.39 × 10^−8^ Wm^−2^ for a single-junction cell^37^ and 25 mWm^−2^ for the blackbody limit setup that corresponds to Fig. 3b^36^. On the other hand, multijunction cells and Landsberg setups for outgoing thermal radiation energy harvesting have yet to be demonstrated. Therefore, there is significant room for improvement in the experimental progress in this area.

Based on the existing works on solar energy harvesting and radiative cooling and emerging efforts for converting outgoing thermal radiation to electricity, there are multiple promising pathways to improve the efficiency of existing energy harvesting systems by combining some of these approaches. For example, for solar energy harvesting systems, reducing the cell temperature by utilizing outer space will directly lead to improved efficiency^24,27,43^. There have been some efforts along this direction with single-junction solar cells^27,45–48^. Our analysis here indicates that the beneficial effect of radiative cooling is much more significant for multijunction cells, solar thermophotovoltaics and the Landsberg setup. Similarly, for outgoing thermal radiation energy harvesting, increasing the cell temperature by utilizing the hot sun will directly lead to improved power generation. Moreover, existing solar energy harvesting systems such as solar panels only utilize the sun and only work during the day. It would be interesting if one could redesign existing solar energy harvesting systems for dual purposes: solar energy harvesting during the day and outgoing thermal radiation energy harvesting at night. For example, one could use solar panels as radiative coolers at night for power generation^36^.

In summary, our results highlight the significant potential of simultaneous energy harvesting from the sun and outer space. The results indicate new fundamental opportunities and provide guidance for improving the efficiency of energy harvesting systems.

## Materials and methods

For each energy harvesting system proposed in the main text, the maximum efficiency is obtained by optimizing the extracted work from the sun and outer space. For example, to compute the maximum efficiency of the multijunction cell combined with radiative cooling (Fig. 2a), we perform a detailed balance and thermal balance analysis by following a procedure similar to Eqs. (1) and (2). Here, the operating voltage of each stack *V*(*E*) is optimized as a function of the photon energy *E*. The work $\dot W$ that can be generated from this setup is:

11 $$\dot W = {\int}_{E_{\mathrm{G}}}^\infty {dEqV(E)\rho \left( E \right)\left( {f \cdot \theta \left( {E,T_{\mathrm{S}},0} \right) - f \cdot \theta \left( {E,T_{\mathrm{c}},V\left( E \right)} \right)} \right)}$$

here, each stack working at a particular energy *E* operates at its optimum voltage *V*(*E*). The temperature of the cell *T*_c_ is determined by its radiative heat exchange:

12 $$P_{{\mathrm{in}}}^{{\mathrm{cell}}} + P_{{\mathrm{in}}}^{{\mathrm{cooling}}} = P_{{\mathrm{out}}}^{{\mathrm{cell}}} + P_{{\mathrm{out}}}^{{\mathrm{cooling}}}$$

here, $P_{{\mathrm{in}}}^{{\mathrm{cell}}} = {\int}_{E_{\mathrm{G}}}^\infty {dE\left( {E - qV(E)} \right)\rho \left( E \right)\left( {f \cdot \theta \left( {E,T_{\mathrm{S}},0} \right)} \right)}$ is the total heat generation in the multijunction cell by absorbing photons from the sun through the restricted solid angle of the sun as described by the view factor *f*. $P_{{\mathrm{in}}}^{{\mathrm{cooling}}} = {\int}_0^\infty {dE\,E\,\rho \left( E \right)\left( {\left( {1 - f} \right) \cdot \theta \left( {E,T_{\mathrm{o}},0} \right)} \right)}$ is the heat received by the bottom radiative cooling layer from outer space at *T*_o_. $P_{{\mathrm{out}}}^{{\mathrm{cell}}} = {\int}_{E_{\mathrm{G}}}^\infty {} dE\left( {E - qV\left( E \right)} \right)\rho \left( E \right)\left( {f \cdot \theta \left( {E,T_{\mathrm{c}},V\left( E \right)} \right)} \right)$ is the total heat dissipation of the cell through re-emission. The cell temperature is *T*_c_, and the voltage of each stack is *V*(*E*). $P_{{\mathrm{out}}}^{{\mathrm{cooling}}} = {\int}_0^\infty {dE\,E\,\rho \left( E \right)\left( {\left( {1 - f} \right) \cdot \theta \left( {E,T_{\mathrm{c}},0} \right)} \right)}$ is the heat dissipation of the bottom radiative cooling layer through thermal radiation.
